# IMPROVING HEALTH CARE OF LGBT OLDER ADULTS: INTERDISCIPLINARY PROJECT ENHANCES HEALTH OUTCOMES

**DOI:** 10.1093/geroni/igz038.2719

**Published:** 2019-11-08

**Authors:** Noell L Rowan, Stephanie D Smith, Tamatha Arms, Kris L Hohn

**Affiliations:** University of North Carolina Wilmington, Wilmington, North Carolina, United States

## Abstract

To date, there is a dearth of interdisciplinary simulation education and research that involves LGBT older adults within schools of social work and nursing. The purpose of this mixed method study was to examine the use of an intervention among social work and nursing students to determine if lecture and simulations impacted their health-related knowledge and cultural sensitivity/awareness of health provisions with LGBT older adults. Interprofessional faculty created lecture and interdisciplinary simulations with actual members of the older LGBT communities using simulation clinic/lab and health care scenarios. An adapted survey with permission from Grubb et al (2013) was used to include quantitative and qualitative measures of cultural awareness with LGBT populations. Pre-Post test data were analyzed using Generalized Linear Models in SAS software. Results indicated that the intervention positively changed perceptions and increased knowledge among (N=90; 32 social work; 58 nursing) allied health students. Statistically significant change experiences in their work with LGBT individuals were noted to positively alter their beliefs about sexuality, gender identity, and sexual development (Agree to Strongly Agree, X2(1)=26.51, p<0.001). Qualitative findings include four primary themes about how gender identity and sexual orientation influences health: (a) bias of health care providers, (b) access to quality care, (c) specific health care needs, and (d) health risks of LGBT older adults. As older adults continue to be the largest population needing health care, it is imperative that professionals are trained to give culturally sensitive health care and demonstrate this competency in their practice and interpersonal interactions with clients.

